# Studying Fluorescence Sensing of Acetone and Tryptophan and Antibacterial Properties Based on Zinc-Based Triple Interpenetrating Metal–Organic Skeletons

**DOI:** 10.3390/molecules28217315

**Published:** 2023-10-28

**Authors:** Congying Yuan, Yidan Qiao, Zhaolei Zhang, Yinhang Chai, Xiaojun Zhang, Xiaojing Dong, Ying Zhao

**Affiliations:** 1 School of Life Science, Luoyang Normal University, 6 Jiqing Road, Luoyang 471934, China; pingboycy@163.com (C.Y.); z2015797093@163.com (X.Z.); 13383799287@163.com (X.D.); 2 Henan Province Function-Oriented Porous Materials Key Laboratory, College of Chemistry and Chemical Engineering, Luoyang Normal University, Luoyang 471934, China; qiaoyidan2023@163.com (Y.Q.); 18339102501@163.com (Z.Z.); chaiyinhangyh@163.com (Y.C.); 3 College of Materials and Chemical Engineering, China Three Gorges University, No. 8, Daxue Road, Yichang 443002, China; 4 College of Chemistry, Zhengzhou University, Zhengzhou 450001, China

**Keywords:** MOFs, antibacterial, fluorescence sensing, acetone, tryptophan

## Abstract

Two triple interpenetrating Zn(II)-based MOFs were studied in this paper. Named [Zn_6_(1,4-bpeb)_4_(IPA)_6_(H_2_O)]_n_ (**MOF-1**) and {[Zn_3_(1,4-bpeb)_1.5_(DDBA)_3_]_n_·2DMF} (**MOF-2**), {1,4-bpeb = 1,4-bis [2-(4-pyridy1) ethenyl]benze, IPA = Isophthalic acid, DDBA = 3,3′-Azodibenzoic acid}, they were synthesized by the hydrothermal method and were characterized and stability tested. The results showed that **MOF-1** had good acid–base stability and solvent stability. Furthermore, **MOF-1** had excellent green fluorescence and with different phenomena in different solvents, which was almost completely quenched in acetone. Based on this phenomenon, an acetone sensing test was carried out, where the detection limit of acetone was calculated to be 0.00365% (volume ratio). Excitingly, the **MOF-1** could also be used as a proportional fluorescent probe to specifically detect tryptophan, with a calculated detection limit of 34.84 μM. Furthermore, the mechanism was explained through energy transfer and competitive absorption (fluorescence resonance energy transfer (FRET)) and internal filtration effect (IFE). For antibacterial purposes, the minimum inhibitory concentrations of **MOF-1** against *Escherichia coli* and *Staphylococcus aureus* were 19.52 µg/mL and 39.06 µg/mL, respectively, and the minimum inhibitory concentrations of **MOF-2** against *Escherichia coli* and *Staphylococcus aureus* were 68.36 µg/mL and 136.72 µg/mL, respectively.

## 1. Introduction

Among many amino acids, tryptophan is one of the amino acids that make up human proteins, and abnormal metabolism of this amino acid can lead to anxiety, dysphagia, hallucinations, and delusions [1,2,3]. In addition, acetone is an important raw material for organic synthesis, and long-term exposure will cause irreversible damage to liver organs or nerves and cause inflammation [4]. Therefore, accurate and rapid detection of tryptophan and acetone are of great significance for disease diagnosis and food nutrition analysis [5]. More worryingly, microbial infection is closely related to human daily life, and such infection may lead to death. Among these infections, bacterial infection is one of the most common infections in hospitals and communities, and it is the main cause of high morbidity and mortality in the world; thus, bacterial infection has attracted extensive attention of scientific researchers [6,7,8,9,10,11]. Hence, it is imperative to develop a material with fluorescence sensing and antibacterial properties.

A metal–organic framework (MOF) is a kind of inorganic–organic coordination hybrid material composed of metal ions or metal clusters linked by organic ligands [12,13]. Because of its high porosity and adjustable structure and function, MOFs have broad application prospects in gas adsorption and separation [14,15,16], catalysis [17,18,19,20], drug delivery [21,22], biological imaging [23,24], chemical sensors [25,26,27], proton conduction [28], and other fields. At the same time, with the rapid development of society, people have put forward higher requirements for environmental protection; however, among these promising properties, fluorescence sensing properties are considered to be an effective method to identify pollutants in the environment [29,30]. For instance, an example of a double-emission Eu-MOF with a specific pH-regulated luminescence conversion characteristic, which can be used as a short-range pH sensor, can be used for the rapid detection of aspartate (Asp) and glutamic acid (Glu) as reported by Li et al. [31]. Furthermore, Zhao and other co-authors reported a case of a three-dimensional metal–organic framework (MOF) {[Eu(L)(H_2_O)_2_]·DMF}_n_ with high solvent, pH, and thermal stability for the first time to detect two amino acids [32]. In addition, it is worth noting that, compared with other disinfectants and antimicrobials, MOFs have major advantages such as high durability, long-term performance, and thermal stability [33]. In the synthesis of antibacterial MOFs, polar organic solvents, soluble salts (Zn^2+^, Co^2+^, Cu^2+^) as metal central sources, and azo compounds as organic linkers make it easy for metal cations to be functionally adjusted in the synthesis of MOFs; these metal cations usually have good antibacterial activity and can be easily introduced into the framework [34]. Therefore, the synthesis of new stable MOF materials with fluorescence sensing properties and antibacterial properties are of great significance.

**Scheme 1:**
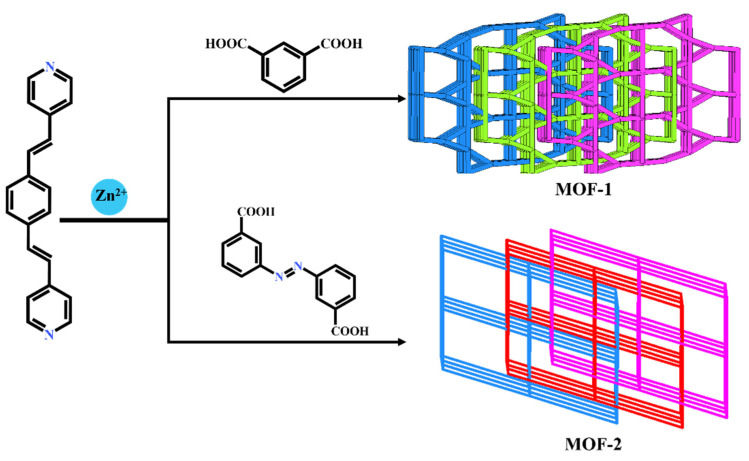
Synthetic protocol of **MOF-1** and **MOF-2** presented in this study.

Based on the above, two triple-interspersed structures, **MOF-1** and **MOF-2,** were designed to be synthesized (Scheme 1). In addition to demonstrating good sensing properties, the MOF material also has high ion release capacity, antibacterial properties, reliable biosafety, and can be used for broad spectrum bacterial sterilization. Here, we report on two functional MOF materials. The structure of the material was characterized by powder X-ray diffraction (XRD), X-ray photoelectron spectroscopy (XPS), scanning electron microscopy (SEM), and Fourier transform infrared spectroscopy (FTIR). The experimental results demonstrated that **MOF-1** had excellent fluorescence properties, so specific sensing tests for tryptophan and acetone could be performed based on the easy and fast response characteristics of the fluorescence spectrum. Meanwhile, the bacteriostatic properties of **MOF-1** and **MOF-2** against *Escherichia coli* and *Staphylococcus aureus* were investigated by bacterial growth curves and plate coating experiments. This work lays a foundation for the development of multifunctional materials with sensing and antibacterial properties.

## 2. Results

### 2.1. Description of the ***MOF-1*** and ***MOF-2*** Crystal Structure

**Figure 1:**
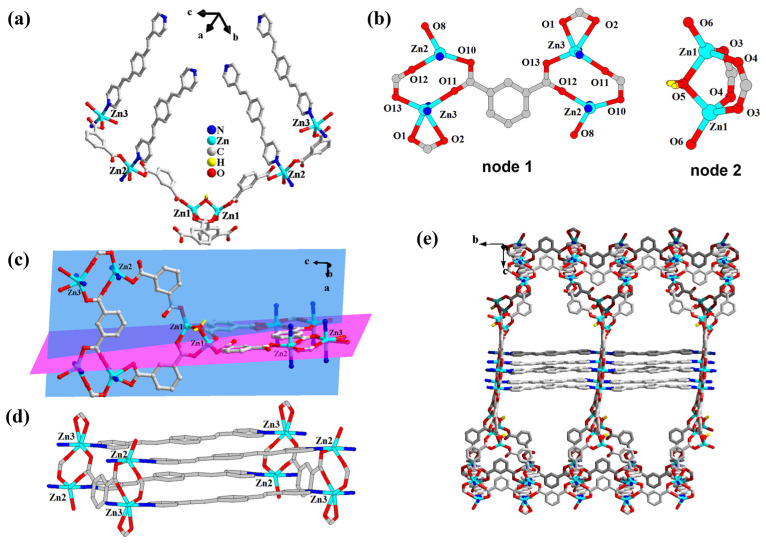
(**a**) View of the **MOF-1** structural unit. (**b**) Two Binuclear Zn-SBUs in **MOF-1**. (**c**) Two types of SBUs are connected to the IPA into a triangle. (**d**) Node 1 extends along the *b*-axis. (**e**)View of the **MOF-1** 3D structure [35].

The crystal structure details of **MOF-1** and **MOF-2** are described below. The structure of **MOF-1** has been previously reported [35]. In this paper, the properties of **MOF-1** are mainly discussed. For detailed structure analysis, please refer to the references cited in the crystal synthesis section. A brief structure diagram of **MOF-1** is shown in Figure 1a–e. The structure description section mainly analyzes **MOF-2**.

**Figure 2:**
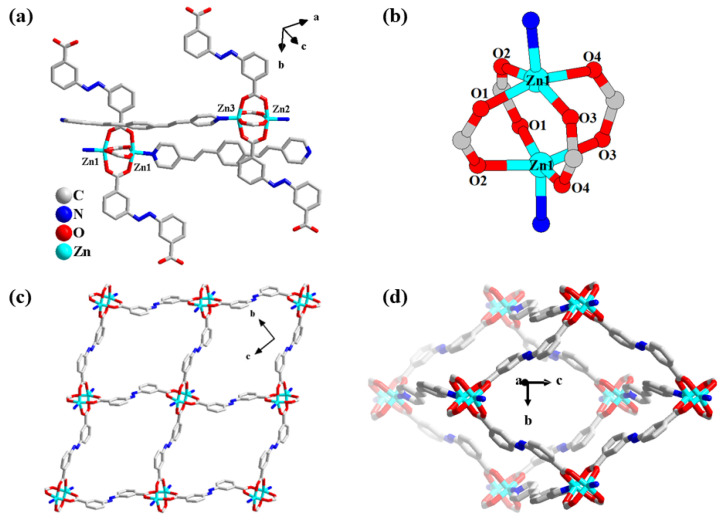
(**a**) View of the **MOF-2** structural unit. (**b**) Binuclear Zn-SBUs of the MOF. (**c**) The 2D network extending along the bc-plane. (**d**) The 3D frame structure of **MOF-2**.

**MOF-2** crystallized in the tetragonal C2/c space group; its structural unit are shown in Figure 2a, which consist of three zinc atoms, one-and-a-half 1,4-bpeb ligands and three DDBA ligands. Although there were two Binuclear Zn-SBUs in the structural unit, they were not connected and interspersed with each other. As such, we took one of them as an example to illustrate the crystal structure. The binuclear Zn-SBUs were composed of two five-coordination Zn1 (Figure 2b), with four oxygen from four DDBA ligands and two N from two 1,4-bpeb ligands. On the bc-plane, the SBUs was connected by DDBA, extending into a two-dimensional plane (Figure 2c). The planes were connected by 1,4-bpeb to form a three-dimensional frame structure (Figure 2d). Finally, the topology structure of the **MOF-2** triple interpenetration was obtained by simplifying the crystal structure (Scheme 1).

### 2.2. Phase Purity and Stability of ***MOF-1*** and ***MOF-2***

**Figure 3:**
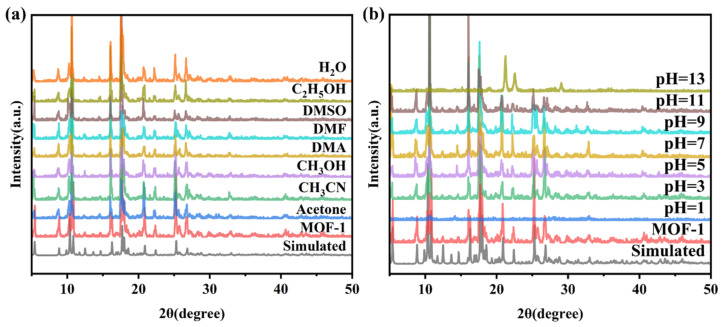
The PXRD patterns of **MOF-1** after different treatments: (**a**) soaked in different solvents, (**b**) soaked in solutions with different pH values prepared by NaOH and HCl.

Firstly, the phase purity of the samples of **MOF-1** and **MOF-2** were verified by comparing the measured PXRD pattern with that obtained by single crystal simulation. The results showed that the experimental peaks were almost identical with the simulated peaks (Figures S1 and S2). It can be seen from Figures S3 and S4 that **MOF-1** had better thermal stability than **MOF-2**. In the temperature range from 25 °C to 380 °C, **MOF-1** had almost no mass loss, while the TGA curve of **MOF-2** had the first mass loss of 10.11% before 315 °C, which may have been due to the increasing temperature, resulting in the loss of guest solvent molecules in the channels. Fortunately, the loss of two DMF guest molecules (10.22%) was found to be close to the value of the first mass loss. Then, at 365 °C, the framework began to collapse with the gradual loss of ligands. Clearly, the thermal stability of **MOF-2** could be maintained at 365 °C. In addition, **MOF-1** was soaked in several common solvents and different pH solutions for 24 h to evaluate its chemical stability. As can be seen from Figure 3a, the intensity and position of PXRD diffraction peaks did not change after **MOF-1** was soaked in different solvents, indicating that **MOF-1** could maintain a stable crystal structure in these media. As can be seen from Figure 3b, the crystal was also very stable in acidic and alkaline aqueous solutions (pH = 3~11).

### 2.3. XPS Analysis

**Figure 4:**
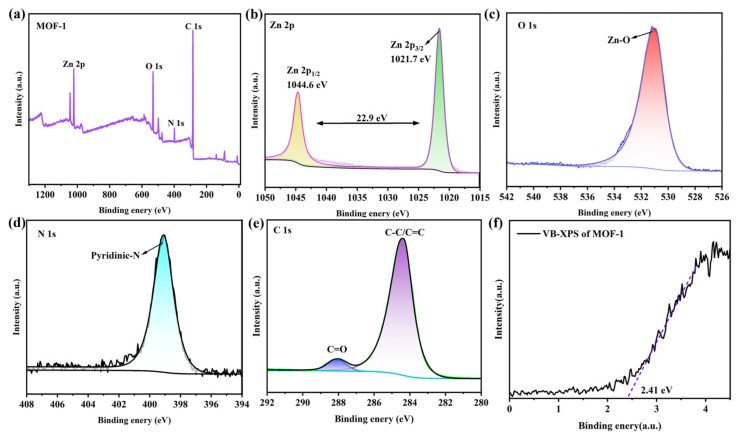
(**a**) the measurement of **MOF-1** and (**b**–**e**) high-resolution XPS spectra; (**f**) VB-XPS spectra of **MOF-1**.

In order to further study the surface chemical composition and valency of the complex, the elements and their chemical composition in **MOF-1** and **MOF-2** were analyzed by XPS. The study spectra of **MOF-1** (Figure 4a) showed that important elements such as C, O, N, and Zn were observed in the resulting complex. In addition, **MOF-1** was studied using high-resolution XPS spectroscopy. As shown in Figure 4b, high-resolution Zn 2p spectra showed that the binding energy of Zn 2p had two peak regions of 1021.7 and 1044.6 eV, corresponding to the spin–orbit splitting of Zn 2p_3/4_ and Zn 2p_1/2_; the binding energy difference between the two was 23 eV, which is consistent with previous reports. The O1s peak at 531.5 eV was characteristic of the Zn-O coordination (Figure 4c). The spectral peak of N1s was shown as pyridinic-N (399.0 eV) (Figure 4d). High-resolution C1s spectra showed two peaks at 284.6 eV and 288.2 eV, corresponding to C=C, C-C, and C=O-OH, respectively (Figure 4e). Furthermore, as shown in Figure 4f, the VB-XPS spectrum further showed that the valence band (VB) of **MOF-1** was analyzed at 2.41 eV [36]. Similarly, we also performed XPS tests on **MOF-2**. The results of the test were similar to **MOF-1** and will not be described here (Figure S5).

### 2.4. SEM and EDS Analysis

**Figure 5:**
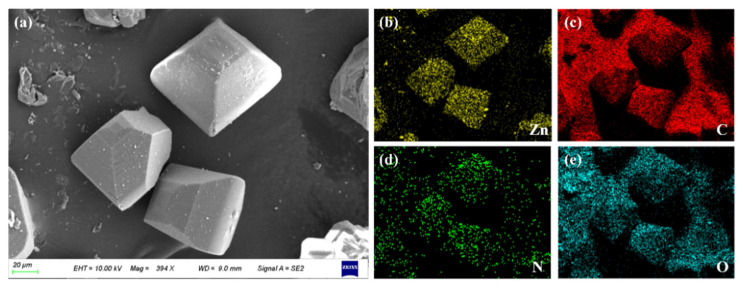
(**a**) SEM images and (**b**–**e**) EDS spectral analysis of **MOF-1**.

The morphology, shape, and size of the **MOF-1** and **MOF-2** samples (Figure 5a and Figure S5) were observed by SEM at an acceleration voltage of 200 eV–30 keV. The three-dimensional structure of the material can be observed with the high resolution of scanning electron microscopy, and the in-depth study was carried out in combination with the element mapping (Figure 5b–e). C, O, N, and Zn elements exist in both **MOF-1** and **MOF-2**. The difference is that **MOF-1** appears as a cube of varying sizes, while **MOF-2** is a three-dimensional parallelogram that is much larger than **MOF-1**.

### 2.5. Solvent Sensing Tests

**Figure 6:**
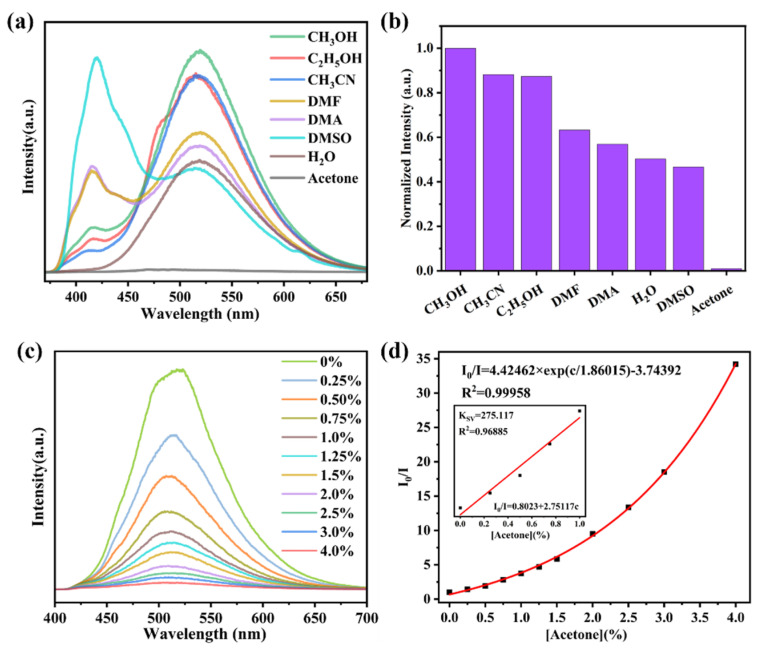
(**a**) The fluorescence spectra of **MOF-1** in different solvents. (**b**) The corresponding peak intensity of **MOF-1** at 510 nm. (**c**) The fluorescence spectrum of **MOF-1** after adding acetone was quantified. (**d**) The plot of *I*_0_/*I* and acetone content in **MOF-1** suspension (Illustrations show data from 0 to 1.0% (volume ratio).

Based on the good green fluorescence of **MOF-1** (Figure S7), its fluorescence phenomena in different solvents were discussed separately. Dispersion of 2 mg **MOF-1** in 3 mL of eight commonly used solvents showed significant differences in the fluorescence spectral shape and intensity; **MOF-1** had almost no fluorescence intensity in acetone under the same conditions (Figure 6a,b). Therefore, the **MOF-1** was titrated with acetone and the detection limit was studied. As shown in Figure 6c, with the addition of acetone, the peak intensity at 510 nm gradually decreased, and the fluorescence intensity was almost zero when 4.0% (volume ratio) acetone was contained in the suspension. Additionally, the relative strength ratio of *I*_0_/*I* increased exponentially with the increase in the acetone content, which can be expressed by the formula *I*_0_/*I* = 4.42462 × exp(*c*/1.86015) − 3.74392 [37]; the correlation coefficient was 0.99958. In the range of 0–1% (volume ratio), the linear growth trend was consistent, and the linear fitting showed that R^2^ = 0.96885, *K*_SV_ = 275.117. Therefore, the detection limit of **MOF-1** toward acetone based on 3σ/*K*_SV_ (σ is standard deviation) [37] was 0.0036% (volume ratio). These results indicate the potential application of the crystal as a material for acetone fluorescence sensing.

### 2.6. Amino Acid Sensing Tests

**Figure 7:**
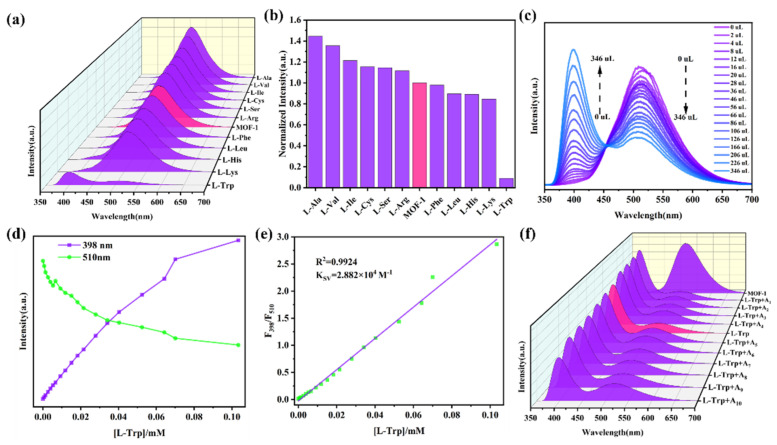
(**a**) Fluorescence spectrum of **MOF-1** in water containing various amino acids. (**b**)The corresponding peak intensity of **MOF-1** at 510 nm. (**c**) The fluorescence spectrum of **MOF-1** after adding L-Trp was quantified. (**d**) Broken line diagram of the luminescent intensity at 398 and 510 nm as a function of the L-Trp concentration. (**e**) Linear correlation between (*F*_398_/*F*_510_) and the L-Trp concentration using **MOF-1**. (**f**) The fluorescence spectra of L-Trp were compared when it was mixed with other ten amino acids.

Due to the excellent water stability and luminescence property (Figure S7) of **MOF-1**, this sparked our interest in studying the sensing performance of an amino acid aqueous solution. Subsequently, eleven kinds of common amino acids were selected, including L-Alanine (L-Ala), L-Valine (L-Val), L-Isoleucine (L-Ile), L-Cysteine (L-Cys), L-Serine (L-Ser), L-Arginine (L-Arg), L-Phenylalanine (L-Phe), L-Leucine (L-Leu), L-Histidine (L-His), L-Lysine (L-Lys), and L-Tryptophan (L-Trp). We added the ground **MOF-1** (2 mg) into the amino acid solution (3 mL, 1 mM), ultrasonicated for 30 min to disperse homogeneously, then recorded the emission spectra. As given in Figure 7a, the emission spectrum of the L-Trp suspension had the most obvious change. Further, L-Trp showed a strong quenching effect, with the quenching efficiency of 91.12% (Figure 7b). In addition, the titration experiments were performed to study the detection limit of **MOF-1**. As shown in Figure 7c, in the aqueous solution of **MOF-1**, the fluorescence intensity at 510 nm was obvious, but almost none at 398 nm. With the addition of L-Trp, the fluorescence at 510 nm gradually decreased, and the fluorescence at 398nm gradually increased (Figure 7d). Additionally, the relative strength ratio of *F*_398_/*F*_510_ increased linearly with the enhancing concentration of antibiotics, and the correlation coefficient was 0.9924. According to the calculated results, the corresponding *K*sv values were 2.882 × 10^4^ M^−1^ for L-Trp (Figure 7e). The detection limit of **MOF-1** toward L-Trp based on 3*σ*/*K*sv [38,39] (σ is standard deviation) was 34.84 μM. However, in practical applications, it is not only required that the sensor has accurate sensitivity, but also required that the sensor has high selectivity for the target material. As given in Figure 7f, when An (A1 = L-Val, A2 = L-Ser, A3 = L-Leu, A4 = L-Arg, A5 = L-Phe, A6 = L-Lys, A7 = L-Ile, A8 = L-Ala, A9 = L-Cys, A10 = L-His) was mixed with L-Trp, the shape and intensity of the fluorescence spectra remained basically unchanged compared with L-Trp, but significantly changed compared with the **MOF-1** suspension. It showed the sensing effect of **MOF-1** on L-Trp was not affected by the other amino acids. All the above results indicate that **MOF-1** has a promising application as a material for L-Trp fluorescence sensing.

### 2.7. Luminescence Sensing Mechanism

In order to elucidate the mechanism of the **MOF-1** sensing process by acetone and L-Trp, we conducted the following spectral studies, where there were two possible interactions: fluorescence resonance energy transfer (FRET) and internal filtration effect (IFE). Good internal filtration effect is manifested in the need for a good spectral overlap between the absorption spectrum of the absorbent and the excitation spectrum of the fluorophore. As shown in Figure S10, **MOF-1** had an excitation band at 275 nm, and L-Trp and acetone had an absorption peak at 250–300 nm, which greatly overlapped the excitation spectrum of **MOF-1**. Thus, tryptophan and acetone shielded the emission of **MOF-1** at 530 nm through competitive absorption [40,41]. However, the FRET mechanism refers to the physical phenomenon of energy being transferred from one excited fluorophore to another. The former is called the donor, the latter is called the acceptor, and FRET occurs when two fluorescent dyes are close enough (when the two molecules interact), and the excited donor transfers part of its energy to the acceptor in a non-radiative manner, so that the acceptor is excited to fluoresce, while its own fluorescence is quenched. As shown in Figure S11, the emission spectrum of tryptophan in water media overlapped greatly with the absorption spectrum of **MOF-1**, which confirmed that the FRET mechanism may exist in proportional fluorescence phenomenon. In addition, in order to gain a deeper understanding of the sensing mechanism, the photoinduced electron transfer (PET) process between the donor and recipient was studied in detail through density functional theory (DFT). From the HOMO-LUMO energy diagram (Figure S11), it can be seen that in the assembly process of **MOF-1**, the light emission was mainly concentrated on 1,4-bpeb, so the DFT of 1,4-bpeb was used to replace **MOF-1**. Furthermore, as can be seen from Figure S11, The HOMO-LUMO gap of L-Trp (5.219 eV) was higher than that of 1,4-bpeb (3.4317 eV). At the same time, the energy of the conduction band of L-Trp (−0.1512 eV) was also higher than that of 1,4-bpeb (−2.3231 eV), which also indicated that the electron transfer of L-Trp to 1,4-bpeb was more favorable. This promoted the transfer of electrons from L-Trp to the lowest occupied orbit of the sensor during photoexcitation, while the luminescence was enhanced. Therefore, the photoinduced electron transfer (PET) sensing mechanism is a cause of enhanced luminescence.

### 2.8. Antibacterial Properties of ***MOF-1*** and ***MOF-2***

**Figure 8:**
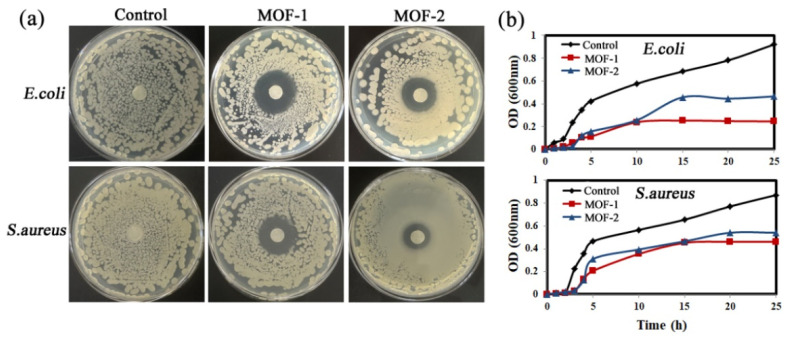
(**a**) Effect of **MOF-1** and **MOF-2** on bacteriostasis of *Escherichia coli* and *Staphylococcus aureus*; (**b**) Bacteriostatic dynamic curve of **MOF-1** and **MOF-2** material.

The results showed (Figure 8a and Figure S13) that in the controlled experiment, except for Zn(NO_3_)_2_·6H_2_O, no antibacterial zone was observed on the AGAR plate, indicating that the other substances did not have antibacterial properties. However, the bacteriostatic zone formed by the metal salts was small and there were speckled colonies, but the two MOF materials had obvious clean bacteriostatic zones on the AGAR plate, indicating that they had a good inhibition effect on *Escherichia coli* and *Staphylococcus aureus* The antibacterial ring of **MOF-1** was significantly larger than that of **MOF-2**, indicating that the antibacterial effect of **MOF-1** on the two bacteria was greater than that of **MOF-2**. The other two materials had different inhibitory effects on *Escherichia coli* and *Staphylococcus aureus*. The inhibitory ring produced by *Escherichia coli* was larger than that produced by *Staphylococcus aureus*, possibly because the cell wall of *Staphylococcus aureus* (Gram-positive bacteria) was thicker than that of *Escherichia coli* (Gram-negative bacteria), which made it more difficult for the material to enter the cells of *Staphylococcus aureus*, as its bacterial structure is not easily destroyed.

In addition, the minimum inhibitory concentration and inhibitory dynamic curve were investigated; the minimum inhibitory concentrations of **MOF-1** against *Escherichia coli* and *Staphylococcus aureus* were 19.52 µg/mL and 39.06 µg/mL, respectively. The minimum inhibitory concentrations of **MOF-2** against *Escherichia coli* and *Staphylococcus aureus* were 68.36 µg/mL and 136.72 µg/mL, respectively. The inhibitory effect of **MOF-1** was higher than that of **MOF-2** from the minimum inhibitory concentration, which was consistent with the results of the inhibitory zone experiment. As can be seen from Figure 8b, the bacteria in both the control group and the experimental group had typical microbial growth stages of a slow stage, logarithmic stage, stable stage, and decline stage. The OD values of the experimental group were significantly lower than those of the control group, indicating that the two materials significantly inhibited the growth of both bacteria at each test point. The results reflect the broad-spectrum antibacterial properties of the two materials [42,43].

## 3. Experiment

### 3.1. Synthesis of [Zn_6_(1,4-bpeb)_4_(IPA)_6_(H_2_O)]_n_ (***MOF-1***) [35]

Zn(NO_3_)_2_·6H_2_O (10.1 mg, 0.034 mmol), 1,4-bpeb (4.8 mg, 0.017 mmol), IPA (2.8 mg, 0.017 mmol), 3 mL DMSO, and 1 mL MeOH were placed in a 10 mL glass bottle with 1 mL 0.1 mM aqueous KOH solution. The glass bottle was then loaded into a Teflon-lined stainless-steel autoclave, which was heated at 100 °C for 72 h. The autoclave was allowed to cool naturally to room temperature to obtain bright yellow octahedral crystals, denoted as **MOF-1**. Elemental analysis (%): calculated for C_128_H_90_N_8_O_25_Zn_6_: C, 60.71%; H, 3.58%; N, 4.43%; found: C, 60.38%; H, 3.62%; N, 4.41%; IR (KBr, cm^−1^): 3028.17 (w), 2919.84 (w), 2364.22 (m), 1617.20 (s), 1554.05 (s), 1381.26 (s), 1207.13 (m), 1029.01 (m), 962.55 (m), 830.96 (m), 755.85 (s), 713.98 (m), 616.95 (m), 551.15 (m), 415.5 7 (w) (Figure S8).

### 3.2. Synthesis of {[Zn_3_(1,4-bpeb)_1.5_(DDBA)_3_]_n_·2DMF} (***MOF-2***)

Zn(NO_3_)_2_·6H_2_O (21.4 mg, 0.072 mmol), 1,4-bpeb (10.2 mg, 0.036 mmol), DDBA (9.7 mg, 0.036 mmol), and 3 mL DMF were placed in a 20 mL glass bottle, which was heated at 100 °C for 24 h. The glass bottle was allowed to cool naturally to room temperature to obtain dark red block crystals, denoted as **MOF-2**. Elemental analysis (%): calculated for C, 59.59%; H, 3.95%; N, 9.80%; found: C, 59.67%; H, 4.00%; N, 9.98%. IR (KBr, cm^−1^): 3054.14 (w), 2930.34 (w), 2842.03 (w), 2349.29 (w), 1614.55 (s), 1400.96 (s), 1211.13 (m), 1072.47 (m), 958.57 (w), 771.22 (m), 682.90 (m), 616.05 (m), 550.02 (m), 449.33 (m) (Figure S9).

### 3.3. Detection of Acetone Test

We added the ground **MOF-1** (2 mg) in different solvents (3 mL) and ultrasonicated for 30 min to disperse homogeneously, then recorded the emission spectra. According to the above method, a series of different suspensions (DMF, CH_3_CN, C_2_H_5_OH, DMA, DMSO, CH_3_OH, H_2_O, and acetone) were prepared, and the selectivity experiment of **MOF-1** was carried out. The performance of **MOF-1** for sensing acetone was also examined by adding acetone with different amounts in suspension

### 3.4. Detection of L-Trp Test

We added the ground **MOF-1** (2 mg) in an amino acid solution (3 mL, 1 mM) or water and ultrasonicated for 30 min to disperse homogeneously, then recorded the emission spectra. According to the above method, a series of amino acid suspensions (L-Alanine (L-Ala), L-Valine (L-Val), L-Isoleucine (L-Ile), L-Cysteine (L-Cys), L-Serine (L-Ser), L-Arginine (L-Arg), L-Phenylalanine (L-Phe), L-Leucine (L-Leu), L-Histidine (L-His), L-Lysine (L-Lys), and L-Tryptophan (L-Trp)) were prepared, and the selectivity experiment of **MOF-1** was carried out. The performance of **MOF-1** for sensing L-Trp was also examined by adding L-Trp with different amounts in suspension. The anti-interference experiments on L-Trp were carried out with the **MOF-1** suspension prepared by mixing L-Trp (1.5 mL, 1 mM) with another amino acid (1.5 mL, 1 mM) as the experimental group and the **MOF-1** suspension prepared by L-Trp (3 mL, 0.5 mM) as the control group.

### 3.5. Antibacterial Performance Test

In this paper, the bacteriostatic effects of **MOF-1** and **MOF-2** were evaluated by the bacteriostatic zone method. In addition, the minimum bacteriostatic concentration and the bacteriostatic dynamic curve of the material were also investigated. Gram-negative bacteria (*Escherichia coli*) and Gram-positive bacteria (*Staphylococcus aureus*) were selected as the research objects, and the antibacterial properties of **MOF-1** and **MOF-2** were determined. The diffusion of the tested sample in the solid culture substrate was used to inhibit the growth of the bacteria around it, and a transparent circular antibacterial zone was formed with the sample as the center.

#### 3.5.1. Preparation of LB Liquid Medium

LB liquid medium: We weighed the peptone, yeast extract, and sodium chloride at 10 g, 5 g, and 10 g, respectively, then fully dissolved with 950 µL dd H_2_O, adjusted the pH to 7.2 with a concentration of 5 mol/L NaOH, and set the volume to 1000 µL.

LB solid medium: Peptone, yeast extract, and sodium chloride were weighed at 10 g, 5 g, and 10 g, respectively, then fully dissolved with 950 µL dd H_2_O, adjusted to pH 7.2 at a concentration of 5 mol/L NaOH, and adjusted to 1000 µL by adding 1.5% AGAR powder.

#### 3.5.2. Preparation of Bacterial Suspension

The strains used were: (inclined) *Escherichia coli* strain—*Escherichia coli* ATCC25922 standard strain, (inclined) *Staphylococcus aureus* ATCC25923 standard strain culture 2nd generation. Plate activated strains were used to inoculate *Escherichia coli* and *Staphylococcus aureus* respectively on the prepared solid medium with inoculation rings. Single colonies were isolated by plate and cultured in a constant temperature drying oven at 37 °C for about 20 h. Then, a single colony was selected with the inoculation ring and dissolved in 1 mL LB medium, and was shaken in a constant temperature oscillating incubator at 37 °C and 250 rpm for 4 h. The final concentration of the diluted bacterial solution was 10^−6^.

#### 3.5.3. Determination of Bacteriostatic Zone of MOFs

First, the complex sample was dissolved in sterile water, and a filter paper with a diameter of 6 mm was dissolved in the solution. Then, 10 µL of the bacterial suspension was absorbed and placed on a glass plate (6 cm in diameter) containing the LB medium. The medium was evenly coated with a triangular glass rod. The soaked filter paper was removed with sterile tweezers and placed vertically on the plate containing the bacteria. Finally, the sealed glass plate was cultured in a constant temperature drying oven at 37 °C. After about 20 h, the size of the transparent antibacterial zone was measured with a vernier caliper, and the average value was taken as the final experimental result.

#### 3.5.4. Determination of Minimum Bacteriostatic Concentration of MOFs

We added 100 µL LB liquid medium to each of the first two rows of the 96-well plate; the we absorbed 100 µL of the MOF material stock and added it to the first hole, and then diluted the drug twice; that is, added the liquid into the first hole and then blew it with a pipette gun (at least 3 times) to fully mix the complex with the medium, then absorbed 100 µL and added it to the second hole, and then blew it to fully mix it with the medium liquid. We also absorbed 100 µL into the third hole and repeated until the last hole; 100 µL of bacterial suspension with a dilution concentration of 10^−6^ was added to each well, and after being thoroughly mixed, the 96-well plate was cultured in a constant temperature drying oven at 37 °C for about 20 h to observe the results, and the absorbance was measured with an enzyme label.

#### 3.5.5. Determination of Bacteriostatic Dynamic Curve of MOFs

LB liquid medium was added with the complex liquid; the final concentration of the MOF material was 1/2 of the minimum bacteriostatic concentration, and the material was incubated in a shaking table at 150 rpm at 37 °C, every 1 h for the first 5 h, and then the absorbance was measured by spectrophotometer every 5 h until 25 h.

## 4. Conclusions

In this paper, two kinds of metal–organic skeleton materials [Zn_6_(1,4-bpeb)_4_(IPA)_6_(H_2_O)]_n_ (**MOF-1**) and [Zn_3_(1,4-bpeb)_1.5_(DDBA)_3_]_n_ (**MOF-2**) with three-layer interpenetration structure were designed and synthesized. **MOF-1** had good stability and fluorescence characteristics, which can not only rapidly and sensitively detect acetone (*LOD* = 0.0036%), but also recognize tryptophan (*LOD* = 34.84 μM). In addition, the antimicrobial properties of **MOF-1** and **MOF-2** were compared. The results showed that the inhibitory zone of **MOF-1** was significantly larger than that of **MOF-2**, and the inhibitory zone of *Escherichia coli* was larger than that of *Staphylococcus aureus*. In addition, the inhibitory zone of **MOF-1** was larger than that of **MOF-2** according to the minimum inhibitory concentration, which was consistent with the inhibitory zone experiment results. The antibacterial dynamic curves also reflected the broad-spectrum antibacterial properties of the two materials. In summary, this paper provides a useful reference for the detection of acetone and amino acids by fluorescence spectroscopy, and also provides considerations for the development of new antibacterial materials.

## Data Availability

Not applicable.

## Supplementary Materials

The following supporting information can be downloaded at: https://www.mdpi.com/article/10.3390/molecules28217315/s1. Refs. [44,45,46,47,48] are cited in the Supplementary Materials.

## Sample Availability

Not available.
