# Supplementary material for: Studying Fluorescence Sensing of Acetone and Tryptophan and Antibacterial Properties Based on Zinc-Based Triple Interpenetrating Metal–Organic Skeletons
Source: Molecules. 2023 Oct 28;28(21):7315. doi: 10.3390/molecules28217315 (PMC10648533; doi:10.3390/molecules28217315)
Supplement: Supplementary file 1 [file molecules-28-07315-s001.zip › molecules-2676517-supplementary.pdf]

Supporting information

Studying Fluorescence sensing of acetone and tryptophan and Antibacterial properties based on zinc-based triple interpenetrating metal-organic skeleton

Congying Yuan [1], Yidan Qiao [2,3], Zhaolei Zhang [2], Yinhang Chai [2,4], Xiaojun Zhang [1], Xiaojing Dong [1] and Ying Zhao [2,*]

[1] School of Life Science, Luoyang Normal University, 6 Jiqing Road, Luoyang 471934, China; pingboycy@163.com (C.Y.); z2015797093@163.com (X.Z.); 13383799287@163.com (X.D.)
[2] Henan Province Function-Oriented Porous Materials Key Laboratory, College of Chemistry and Chemical Engineering, Luoyang Normal University, Luoyang 471934, China; qiaoyidan2023@163.com (Y.Q.); 18339102501@163.com (Z.Z.); chaiyinhangyh@163.com (Y.C.)
[3] College of Materials and Chemical Engineering, China Three Gorges University, No. 8, Daxue Road,
Yichang 443002, China
[4] College of Chemistry, Zhengzhou University, Zhengzhou 450001, China
* Correspondence: zhaoying_909@sina.com

Materials and instrumentation

All chemicals and solvents were commercially available and used without any refinement. Powder X-ray diffraction (PXRD) measurements were performed on a Bruker D8-ADVANCE X-ray diffractometer with monochromatic Cu Kα radiation (λ = 1.5406 Å). Elemental analyses for carbon, hydrogen, and nitrogen atoms were performed on a Vario EL III elemental analyzer. Thermogravimetric analysis (TGA) was tested from room temperature to 800 ℃ at a heating rate of 10 ℃/min on a TG/DTA6300 thermal analyzer. The ultraviolet-visible (UV-vis) adsorption spectra were recorded by a PerkinElmer Lambda 950 spectrophotometer at room temperature. Field Emission Scanning electron microscope (FE-SEM) images were obtained from a Hitachi-S4800 scanning electron microscope at an accelerating voltage of 20.0 kV with an energy dispersive X-ray (EDX) spectrometer. X-ray photoelectron spectroscopic (XPS) analysis was carried out on a Kratos AXIS Ultra DLD spectrometer (Kratos Analytical-A Shimadzu) by using a monochromatic Al Kα X-ray source under hybrid magnification mode. Fourier transform infrared spectra (FT-IR) were collected on a Nicolet 6700 Fourier Transform infrared spectrometer. Fluorescent analyses were carried out on a Hitachi F-7000 analyzer. Crystal data for **MOF-1** and **MOF-2** could be found from Oxford SuperNova diffractometer with molybdenum target. The crystal structure was solved by SHELXS-2014. CCDC: 2299318 (**MOF-2**)

Table S1 Crystallographic data for **MOF-1**[1] and **MOF-2**.

Complex	**MOF-1**	**MOF-2**
Empirical formula	$C_{128}H_{90}N_8O_{25}Zn_6$	$C_{78}H_{62}N_{11}O_{14}Zn_3$
Formula mass	2532.29	1573.56
Crystal system	Tetragonal	Monoclinic
Space group	$P4_32_12$	$C2$/c
a/Å	20.0210(4)	40.462(2)
b/Å	20.0210(4)	18.2148(8)
c/Å	28.1490(10)	22.2158(9)
α/°	90	90
β/°	90	90.705(2)
γ/°	90	90
Volume/Å^3	11283.3(6)	16371.9(13)
Z	4	8
ρ_{calc}g/cm^3	1.491	1.158
Reflections collected	183696	77787
F(000)	5176.0	5832.0
μ/mm^{-1}	1.335	0.929
Goodness-of-fit on F^2	1.105	1.046
	R_1=0.1049	R_1=0.0557
R indices [I > 2δ(I)]	wR_2=0.1942	wR_2=0.1462

Table S2. Selected bond lengths (Å) and bond angles (°) for **MOF-1**and **MOF-2**.

MOF-1

Atoms	Lengths (Å)	Atoms	Lengths (Å)
Zn (1)-O (3)	1.975	Zn (1)-O (4)	2.012
Zn (1)-O (5)	1.954	Zn (1)-O (6)	1.939
Zn (2)-O (8)	2.083	Zn (2)-O (10)	2.118
Zn (2)-O (12)	2.164	Zn (3)-O (1)	2.122
Zn (3)-O (2)	2.444	Zn (3)-O (13)	2.093
Zn (3)-O (11)	2.370		
Atoms	Angles (°)	Atoms	Angles (°)
O (3)-Zn (1)-O (4)	101.33	O (3)-Zn (1)-O (5)	104.89
O (4)-Zn (1)-O (5)	101.91	O (12)-Zn (2)-O (10)	133.22
O (8)-Zn (2)-O (12)	90.60	O (2)-Zn (3)-O (11)	89.99
O (11)-Zn (3)-O (13)	123.70	O (1)-Zn (3)-O (13)	89.91

MOF-2

Atoms	Lengths (Å)	Atoms	Lengths (Å)
Zn (1)-O (1)	2.040	Zn (1)-O (2)	2.038
Zn (1)-O (3)	2.051	Zn (1)-O (4)	2.036
Atoms	Angles (°)	Atoms	Angles (°)
O (1)-Zn (1)-O (2)	87.97	O (1)-Zn (1)-O (3)	86.62
O (2)-Zn (1)-O (4)	89.76	O (3)-Zn (1)-O (4)	88.44

Table S3 Comparison of the detection of amino acids by MOFs

Number	Amino acid	LODs(μM)	Reference
1	Aspartic acid	0.26	[2]
2	Aspartic acid	0.46	[3]
3	Aspartic acid	59.1	[4]
4	Histidine	406	[4]
5	Tryptophan	167	[5]
6	Tryptophan	0.0429	[6]
7	Homocysteine	0.04	[7]
9	Tryptophan	34.84	This work

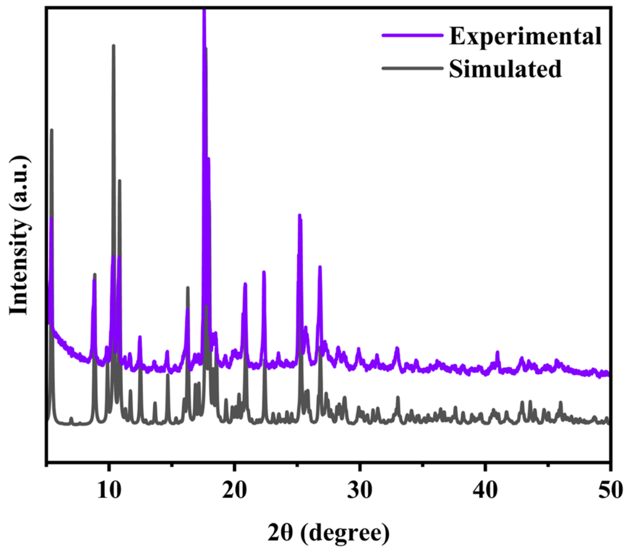

Figure S1. The PXRD patterns of simulated and experimental for **MOF-1**

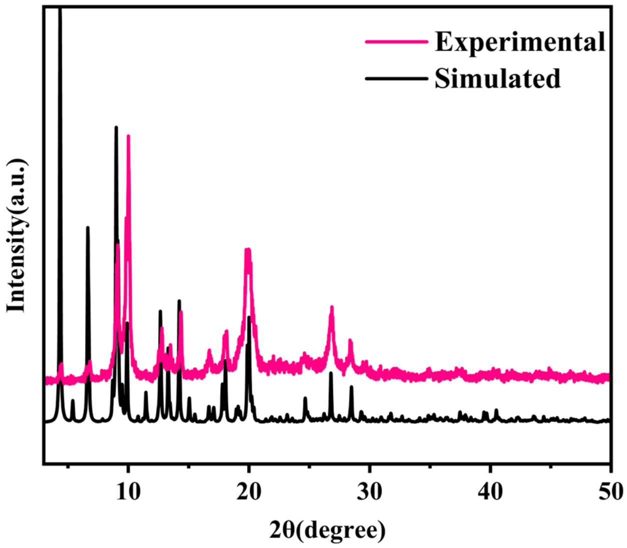

Figure S2. The PXRD patterns of simulated and experimental for **MOF-2**

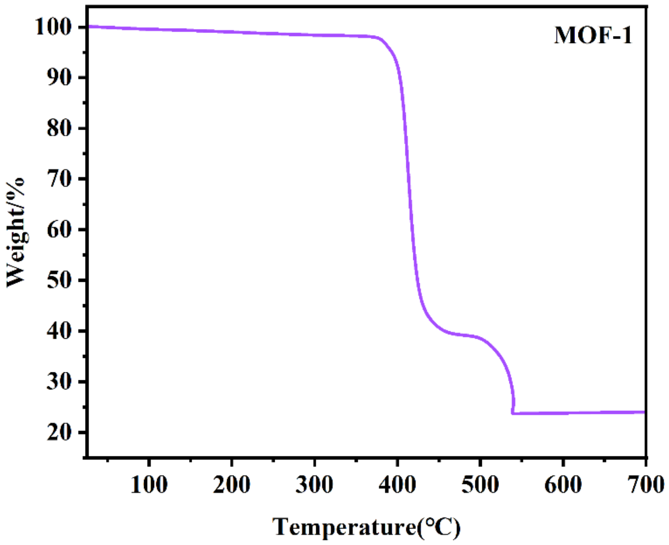

Figure S3. The TGA curve for **MOF-1**

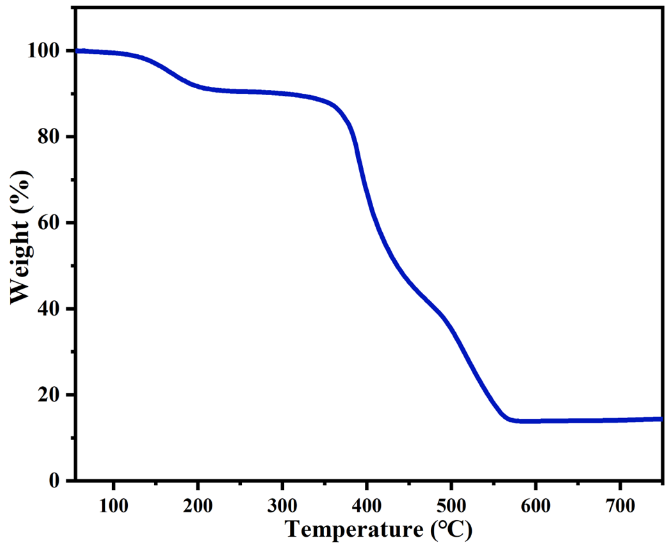

Figure S4. The TGA curve for **MOF-2**

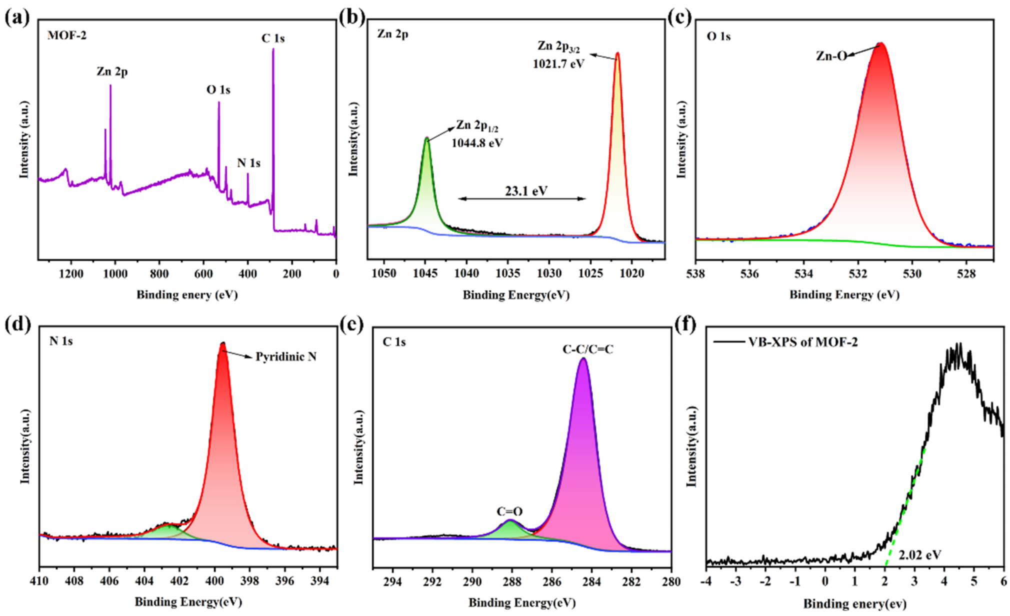

Figure S5 (a) the measurement of **MOF-2** and (b-e) high-resolution XPS spectra; (f) VB-XPS spectra of **MOF-2**

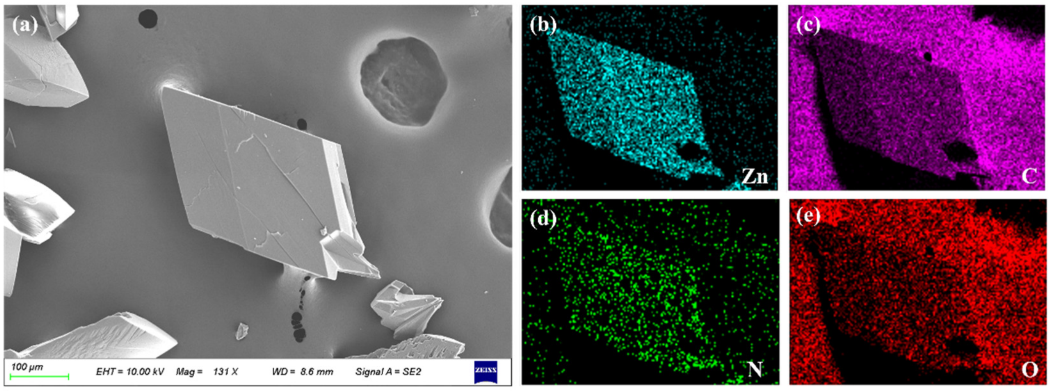

Figure S6 SEM images and EDS spectral analysis of **MOF-2**.

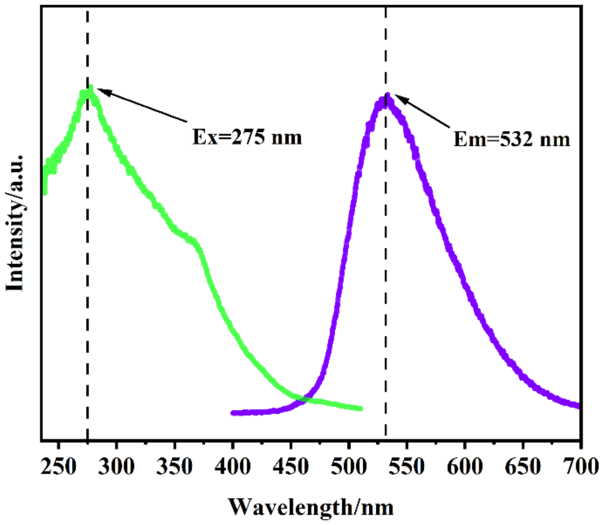

Figure S7 Fluorescence spectra of **MOF-1** solids at room temperature.

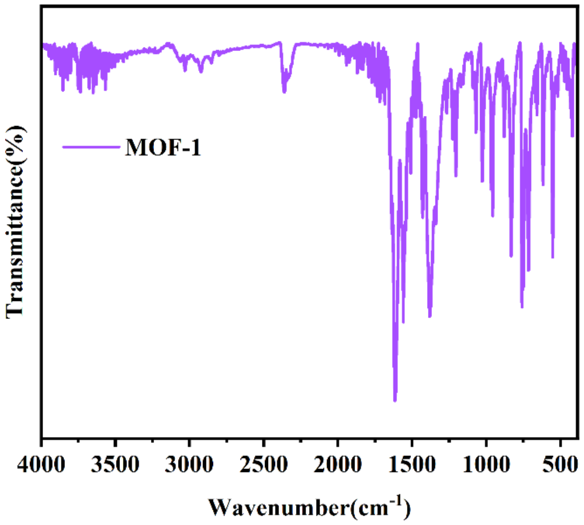

Figure S8 FT-IR spectra of **MOF-1**.

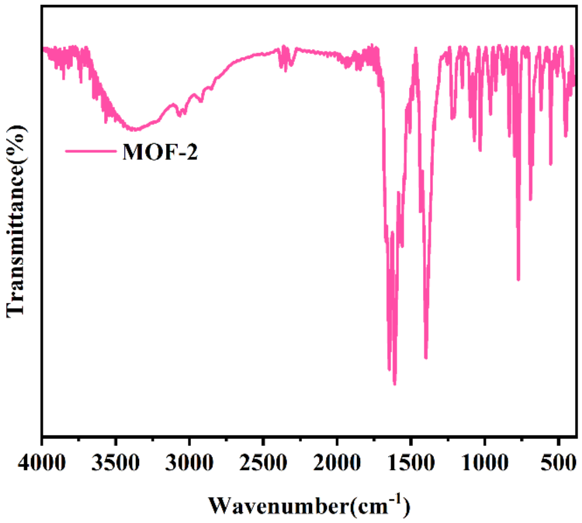

Figure S9 FT-IR spectra of **MOF-2**.

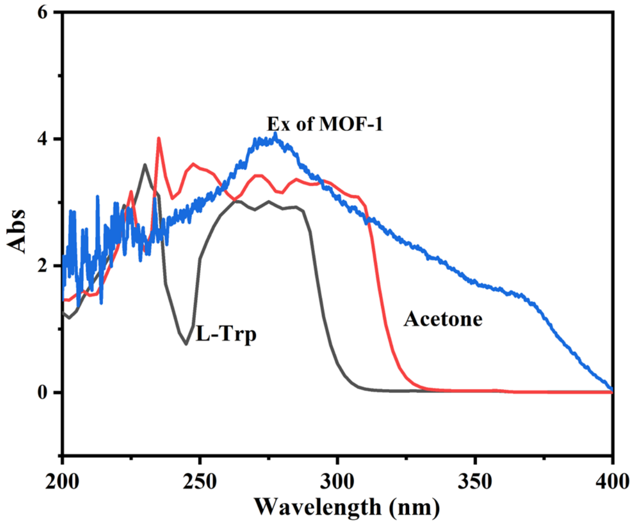

Figure S10. UV–vis spectra of analyte and excitation spectra of **MOF-1**.

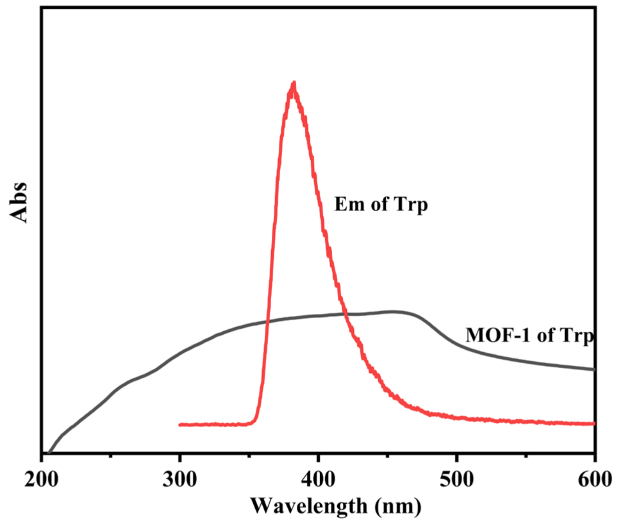

Figure S11. UV–vis spectra of **MOF-1** and emission spectra of L-Trp.

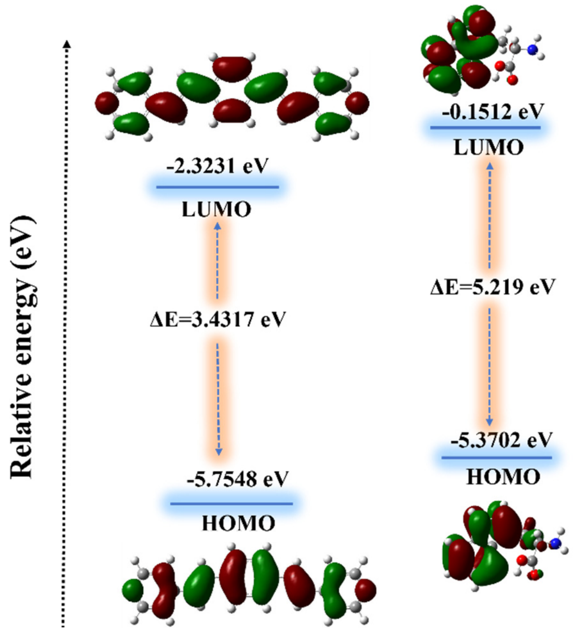

Figure S12. Schematic diagram of the LUMO and HOMO energies of 1, 4-bpeb and L-Trp

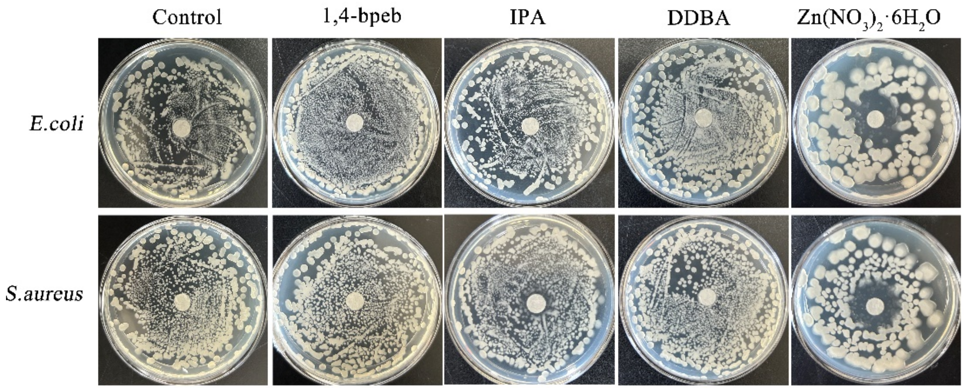

Figure S13. (a) Effect of 1, 4-bpeb, IPA, DDBA and $Zn(NO_3)_2 \cdot 6H_2O$ on bacteriostasis of *Escherichia coli* and *Staphylococcus aureus*

References

[1] Liu, D.; Ren, Z.; Li, H.; Chen, Y.; Wang, J.; Zhang, Y.; and Lang, J.; pH-dependent solvothermal formation of two different 3D multiple interpenetrating nets from the same components of $Zn(NO_3)_2$, 1,3-benzenedicarboxylate and 1,4-bis[2-(4-pyridyl)ethenyl]benzene. CrystEngComm, **2010**, 12, 1912–1919.

[2] Wu, P.; Jiang, M.; Hu, X.; Wang, J.; He, G.; Shi,; Li,; Liu, W.; Wang, J. Amide-containing luminescent metal-organic complexes as bifunctional materials for selective sensing of amino acids and reaction prompting. RSC Adv. **2016**, 6, 27944-27951.

[3] Weng, H.; Yan, B. A sliver ion fabricated lanthanide complex as a luminescent sensor for aspartic acid. Sensor Actuat B-Chem. **2017**, 253, 1006-1011.

[4] Yang, A.; Hou, S.; Shi, Y.; Yang, G.; Qin, D.; and Zhao, B.; Stable Lanthanide-Organic Framework as Luminescent Probe to Detect Both Histidine and Aspartic Acid in Water. Inorg. Chem. **2019**, 58, 9, 6356–6362.

[5] Abdelhamid, H.; Bermejo-Gomez, A.; Martin-Matute, B.; Zou, X. A water-stable lanthanide metal-organic framework for fluorimetric detection of ferric ions and tryptophan. Microchim. Acta. **2017**, 184, 3363-3371.

[6] Zhang, J.; Huang, Y.; Yue, D.; Cui, Y.; Yang, Y.; Qian, G. A luminescent turnup metal-organic framework sensor for tryptophan based on singlet-singlet Förster energy transfer. J. Mater. Chem. B. **2018**, 6, 5174-5180.

[7] Wang, J.; Liu, Y.; Jiang, M.; Li, Y.; Xia, L.; Wu, P. Aldehyde-functionalized metalorganic frameworks for selective sensing of homocysteine over Cys, GSH and other natural amino acids. Chem. Commun. **2018**, 54, 1004-1007.
